# WE’RE NEVER TALKING ABOUT IT: POLICY AND PRACTICE ABOUT END OF LIFE IN ASSISTED LIVING

**DOI:** 10.1093/geroni/igz038.1657

**Published:** 2019-11-08

**Authors:** Alexis A Bender, Ann E Vandenberg, Candace L Kemp, Elisabeth O Burgess, Molly M Perkins

**Affiliations:** 1 Emory University, Atlanta, Georgia, United States; 2 Georgia State University, Atlanta, Georgia, United States

## Abstract

Increasing numbers of people are aging in place and dying in assisted living (AL) and AL is increasingly becoming a site of end-of-life care in the US. Consequently, AL staff frequently experience death and decline of the residents they care for. Using data from ethnographic observations and in-depth qualitative interviews with 14 direct care workers and 16 administrators participating in a 5-year NIA-funded study (R01AG047048) examining end-of-life care in four diverse ALs, we used thematic analysis to examine how AL administrators and staff implement and understand policies and norms around death and dying (e.g., training or use of advance care directives). We found there is limited training regarding death and bereavement and limited formal grief support for staff. These findings identify gaps in communication between administrators and direct care workers with implications for increasing communication about death and dying and improving support for direct care workers experiencing grief and bereavement.

